# Integrated Role of NRF2 and p53 Gene Expression in Hormonal and Biochemical Dysregulation Among Infertile Males

**DOI:** 10.5935/1518-0557.20260010

**Published:** 2026

**Authors:** Aswathy Sundaresh, J S Jiju, Arun William, Aswathi Rajan, Soumya Jose, G Aswathy, Aksa Simon, Vineetha Vijayan, Bobby Joseph, E V Ajitha, Sreeshma P Pillai, D Dinesh Roy

**Affiliations:** 1 Associate Professor, Medical Biochemistry, Sree Gokulam Medical College & Research Foundation, Venjaramoodu, Thiruvananthapuram, Kerala, India; 2 Technical Officer, Division of Pathology, Regional Cancer Centre (RCC), Thiruvananthapuram, Kerala, India; 3 Associate professor, Mount Zion Medical College, Chayalod P.O, Adoor, Kerala, India; 4 Assistant professor, SUT Academy of Medical Sciences, Vencod, Vattappara, Thiruvananthapuram, Kerala, India; 5 Lab Director, Genetika, Centre for Advanced Genetic Studies, Thiruvananthapuram, Kerala, India; 6 Research Coordinator, Genetika, Centre for Advanced Genetic Studies, Thiruvananthapuram, Kerala, India; 7 Research Scholar, Shri Jagdishprasad Jhabarmal Tibrewala University, Vidyanagari, Churela, Rajasthan, India; 8 Research Scholar, Meenakshi Academy of Higher Education and Research (MAHER- Deemed to be University), West K.K Nagar, Chennai, Tamil Nadu, India; 9 CEO & Senior Cytogeneticist, Genetika, Centre for Advanced Genetic Studies, Thiruvananthapuram, Kerala, India

**Keywords:** male infertility, NRF2, p53, oxidative stress, hormonal imbalance, biochemical markers, gene expression

## Abstract

**Objective:**

Male infertility is a multifactorial condition often linked to oxidative stress and apoptosis. Key molecular regulators, such as NRF2 (antioxidant defense) and p53 (cell cycle and apoptosis), are influenced by hormonal and inflammatory pathways; however, their combined role in infertility remains underexplored. This study investigates the integrated expression of NRF2 and p53 genes and their association with hormonal, inflammatory, and biochemical markers in infertile males.

**Methods:**

A case-control study was conducted on 300 males (150 infertile, 150 fertile controls). Gene expression (NRF2, p53) was analyzed using RT-PCR. Hormonal (FSH, testosterone, estradiol), inflammatory (IL-6), and biochemical (SOD, PSA) markers were measured via ELISA. Statistical analysis included t-tests, ROC, scatter plot, and multiple regression.

**Results:**

Infertile men showed significantly reduced NRF2 expression and SOD levels, with elevated FSH, estradiol, IL-6, PSA, and p53 expression (p<0.001). ROC analysis identified FSH and NRF2 as strong predictors of infertility. Regression revealed IL-6 and PSA as significant positive predictors of p53 expression, while SOD positively correlated with NRF2. Scatterplots highlighted contrasting biomarker associations for NRF2 and p53.

**Conclusions:**

Combined dysregulation of NRF2 and p53, driven by oxidative, hormonal, and inflammatory factors, plays a critical role in male infertility. These genes hold promise as both diagnostic biomarkers and therapeutic targets.

## INTRODUCTION

Infertility is a disease of the male or female reproductive system defined by the failure to achieve a pregnancy after 12 months or more of regular unprotected sexual intercourse (World Health Organization, 2023). The etiology of male infertility is multifactorial, encompassing genetic, anatomical, hormonal, and biochemical disruptions. Among the molecular pathways increasingly recognized for their relevance are oxidative stress regulation and apoptosis, which directly influence germ cell survival and function (Agarwal *et al*., 2014).

Oxidative stress is a prominent pathological mechanism in male infertility. The redox-sensitive transcription factor nuclear factor erythroid 2-related factor 2 (NRF2) orchestrates the cellular antioxidant defense by regulating genes involved in detoxification and reactive oxygen species (ROS) neutralization (Yamamoto *et al*., 2018). NRF2 activity is essential for maintaining the redox balance within germ cells, and its downregulation has been associated with reduced sperm motility, DNA fragmentation, and decreased fertilization capacity (Rehman *et al*., 2015).

Conversely, the tumor suppressor gene p53 is a key regulator of cellular responses to genotoxic stress, including DNA repair, cell cycle arrest, and apoptosis (Li *et al*., 2024). While p53 plays a protective role against DNA damage, its overexpression in germ cells has been linked to excessive apoptosis, poor semen quality, and spermatogenic failure (Rahbar *et al*., 2017; Alizadeh *et al*., 2023). Disruption in p53 function can also elevate the risk of reproductive tract malignancies, compounding the clinical burden in infertile men (Tvrda *et al*., 2015).

Significantly, both NRF2 and p53 expression are influenced by the hormonal and biochemical milieu. Hormonal imbalances, such as elevated FSH, LH, and estradiol, or decreased testosterone, have been implicated in defective spermatogenesis and may modulate the NRF2 and p53 pathways. Inflammatory cytokines like interleukin-6 (IL-6) further exacerbate testicular dysfunction by promoting oxidative stress and apoptosis, potentially through interactions with NRF2 and p53 (Hassan *et al*., 2024).

While several studies have independently examined the role of oxidative stress (via NRF2) and apoptosis (via p53) in male infertility, there is limited integrative research evaluating the concurrent expression of these genes in the context of endocrine and inflammatory dysfunction. The present study bridges this gap by investigating the combined gene expression patterns of NRF2 and p53, as well as their association with hormonal (FSH, estradiol, testosterone), inflammatory (IL-6), and biochemical (PSA, SOD) markers in infertile males. By doing so, this research aims to offer a holistic molecular perspective on male infertility and identify potential diagnostic and therapeutic targets that integrate oxidative and apoptotic regulatory networks.

## MATERIALS AND METHODS

### Study Design and Participants

This study employed a case-control design involving 300 male participants, comprising 150 infertile men as cases and 150 proven fertile men as controls. Participants were between the ages of 25 and 45 years. Infertility among the cases was diagnosed according to World Health Organization (WHO) criteria, defined as the inability to achieve conception after at least 12 months of regular, unprotected sexual intercourse. Fertile controls were recruited based on their confirmed ability to father at least one child. All participants were recruited from fertility clinics and diagnostic centres located in Kerala. Ethical clearance for the study was obtained from the Institutional Ethics Committee of Genetika (Reference numbers: 02/2022/IECG), and informed written consent was obtained from each participant before sample collection.

### Inclusion and Exclusion Criteria

Participants with chronic illnesses, malignancies, recent infections, or those who had undergone radiation or chemotherapy were excluded from the study. Additional exclusion criteria included incomplete clinical data or refusal to provide informed consent. Eligible participants included men aged 25-45 years, with infertile cases meeting clinical criteria for primary infertility and controls being men with at least one biological child.

### Sample Collection

Fasting venous blood samples (8-10 mL) were collected from each participant using sterile techniques. Blood was drawn into plain tubes for serum separation and into EDTA-coated tubes for RNA extraction. The serum was isolated by centrifugation and stored at appropriate temperatures for further biochemical and hormonal assays. RNA was extracted from EDTA blood using a commercial RNA extraction kit, and the purity and concentration were determined using a biospectrophotometer.

### Hormonal and Biochemical Analysis

Biochemical and hormonal markers were analyzed using the enzyme-linked immunosorbent assay (ELISA) technique. The hormonal profile included measurements of testosterone, follicle-stimulating hormone (FSH), and estradiol. Inflammatory status was assessed by evaluating interleukin-6 (IL-6) levels. Additionally, superoxide dismutase (SOD) and prostate-specific antigen (PSA) concentrations were determined using ELISA (Origin Diagnostics and Research, Ernakulam, Kerala, India).

### Gene Expression Analysis

Total RNA was extracted using an RNA extraction kit and quantified using a biospectrometer. Reverse transcription was carried out using 50 µg of total RNA with Oligo (dT)18, random hexamers, and reverse transcriptase (RTase), and the resulting cDNA was diluted to 50 ng/µL and stored at -20°C. Gene expression analysis for *p53* and *Nrf2* was performed using Real-Time PCR (RT-PCR) on the CFX Opus 96 Real-Time PCR system, with GAPDH as the reference gene.

For *p53*, the primers used were: Forward: 5’-CCTCAGCATCTTATCCAGTGG-3’ and Reverse: 5’-TGGATGGTGGTACAGTCAGAGC-3’ (each 22 bp; GC%: 54.55; Tm: 62.12°C). For *Nrf2*, the primers were: Forward: 5’-CACATCCAGTCAGAAACCAGTGG-3’ and Reverse: 5’-GGAATGTCTGCGCCAAAAGCTG-3’. Each reaction was prepared in a 20 µL volume containing 2X Real-Time PCR Master Mix, gene-specific primers, cDNA, and nuclease-free water.

The thermal cycling conditions included an initial denaturation at 95°C for 5 minutes, followed by 30-40 cycles of denaturation at 94-95°C for 1 minute, annealing at gene-specific temperatures (54°C for *p53*, 57°C for *Nrf2*) for 1 minute, and extension at 72°C for 1 minute. A final extension step at 72°C for 10 minutes was performed, followed by melt curve analysis to confirm specificity. Relative gene expression was calculated using the 2^(-∆∆Ct) method.

### Statistical Analysis

All statistical analyses were performed using JAMOVI version 2.5.3 and Stata 17.0 software. Descriptive statistics were used to summarize baseline characteristics. Independent t-tests or Mann-Whitney U tests for continuous variables, based on data distribution. Receiver Operating Characteristic (ROC) curve analysis was conducted to assess the diagnostic accuracy of key biomarkers, with area under the curve (AUC), sensitivity, specificity, and cut-off values reported. Regression models were employed to explore the relationship between gene expression levels and clinical or hormonal variables. A *p*-value of less than 0.05 was considered statistically significant throughout all analyses.

## RESULTS

**Figure 1:**
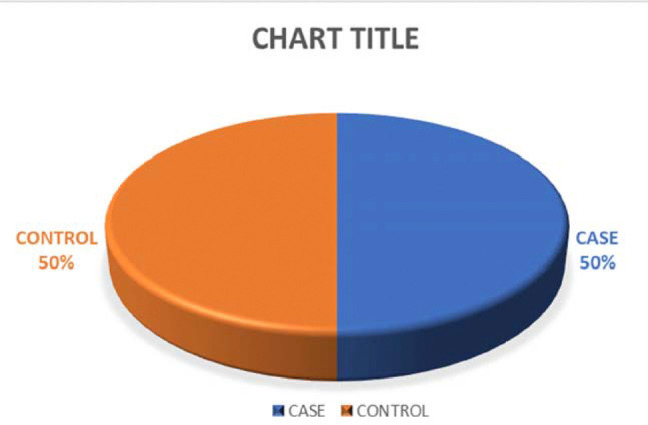
Distribution of study participants (case *vs*. controls).

The study included (Figure 1) 300 participants (aged 25-45), 150 of whom were infertile males and 150 of whom were healthy controls. The participants were selected based on predefined inclusion and exclusion criteria.

Independent samples t-test (Table 1) showed that infertile males had significantly lower SOD levels (*p*<0.001) and NRF2 gene expression (*p*<0.001), along with higher FSH levels (*p*<0.001) compared to controls.

**Table 1:** Independent samples t-test results.

	Control (n=150)		Case (n=150)		t	p
	mean	SD	mean	SD		
SOD (U/mL)	4.10	2.60	2.48	2.28	5.745	0.001
FSH (mIU/mL)	8.72	3.06	16.25	4.73	-16.337	0.001
Nrf 2 gene expression (2^-∆∆CT method)	1.002	0.178	0.600	0.324	13.313	0.001

Data are presented as mean ± standard deviation (SD). Comparison between control (n=150) and case (n=150) groups was performed using independent sample t-test. Statistically significant differences were observed in superoxide dismutase (SOD) activity (U/mL), follicle-stimulating hormone (FSH, mIU/mL), and Nrf2 gene expression levels (2^-∆∆CT), with *p*<0.001 for all parameters, as shown in Table 1.

Table 2 revealed significant differences in hormonal and inflammatory markers between the groups. Infertile males had lower testosterone and higher estradiol, PSA, IL-6, and p53 gene expression levels (all *p*<0.001), indicating hormonal imbalance, inflammation, and increased apoptotic activity.

**Table 2:** Comparative analysis of biochemical and hormonal parameters be tween cases and controls.

Variables	p-value
Testosterone	<0.001
Estradiol	<0.001
Prostate Specific Antigen (PSA)	<0.001
IL-6	<0.001
P53 gene mutation	<0.001

Based on independent sample t-test (Unequal variance), all other p-values based on Mann-Whitney U test.

**Figure 2:**
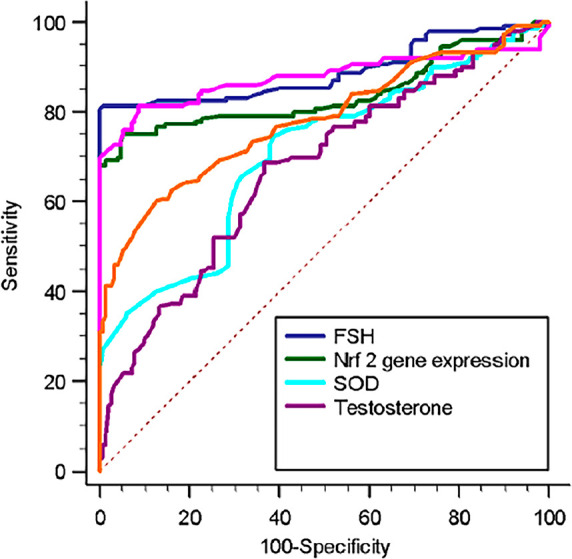
ROC curve analysis. These findings, as visualized in Figure 1, highlight the clinical relevance of hormonal markers, particularly FSH and testosterone, in assessing male reproductive health.

ROC (Figure 2) showed that FSH and Nrf2 gene expression are the strongest predictors of male infertility, with high AUC values and favorable sensitivity, specificity, and likelihood ratios, making them effective indicators of male infertility. Testosterone and SOD have limited diagnostic utility, providing moderate association but weaker discrimination between infertile and non-infertile men. These findings underscore the multifactorial nature of male infertility, with FSH and Nrf2 expression emerging as key biomarkers.

**Figure 3:**
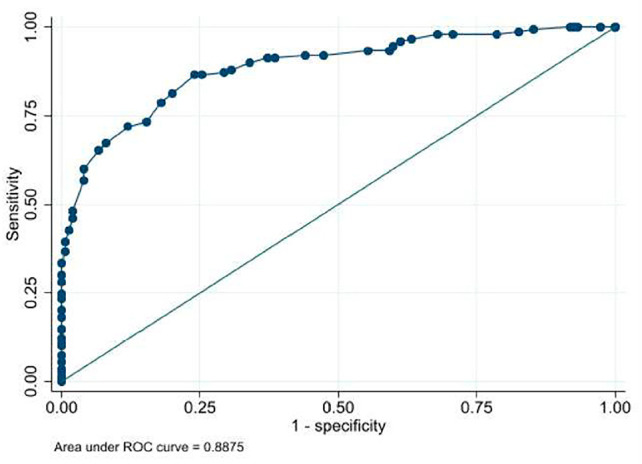
ROC curve for Estradiol for predicting the cases. The predictive performance of the biomarker was further supported by the ROC analysis (Figure 2), which revealed an AUC value close to 0.89.

In Figure 3, the Area Under the Curve (AUC) is 0.8875, indicating that Estradiol is an effective marker for distinguishing between individuals with and without infertility. The Z statistic of 2.35 and the significance level of *p*<0.0001 suggest that the AUC is significantly different from 0.5, demonstrating the reliability of Estradiol in differentiating between the two groups.

**Figure 4:**
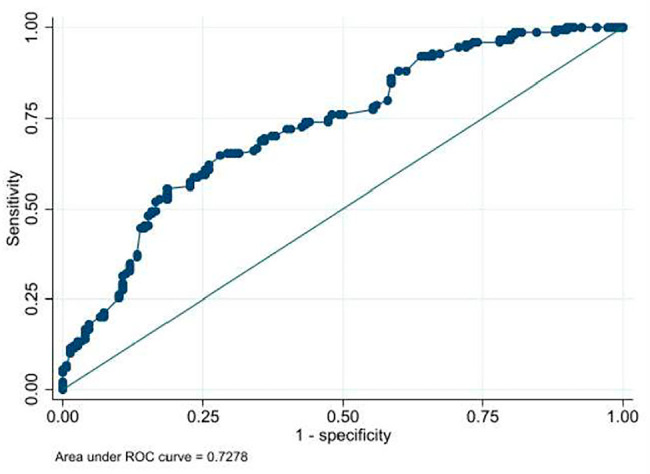
ROC curve for PSA for predicting the cases. According to the ROC curve in Figure 3, the biomarker provides fair classification accuracy between cases and controls.

Figure 4 presents the results of the ROC analysis for Prostate Specific Antigen (PSA) in predicting the cases of prostate-related conditions. The Area Under the Curve (AUC) is 0.7278, which indicates that PSA has a moderate ability to differentiate between individuals with and without the condition. The Z statistic of 3.56 and the significance level of *p*<0.0001 suggest that the AUC is significantly different from 0.5, indicating that PSA is a reliable test for distinguishing between the two groups.

**Figure 5:**
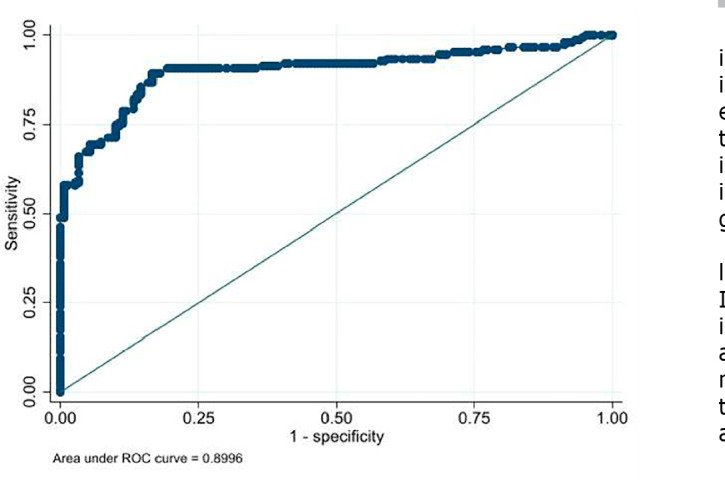
ROC curve for IL-6 for predicting the cases. The ROC analysis presented in Figure 4 demonstrates excellent diagnostic performance of the evaluated biomarker, with an area under the curve (AUC) of 0.8996, indicating high sensitivity and specificity in distinguishing between case and control groups.

Figure 5 presents the results of the ROC analysis for Interleukin-6 (IL-6) in predicting the cases. The Area Under the Curve (AUC) is 0.8996, which indicates that IL-6 is an excellent test for distinguishing between individuals with and without the condition, as the AUC is significantly higher than 0.5. The Z-statistic of 8.51 and the significance level of *p*<0.0001 confirm that the AUC is statistically significant, indicating that IL-6 can effectively differentiate between the two groups.

**Figure 6:**
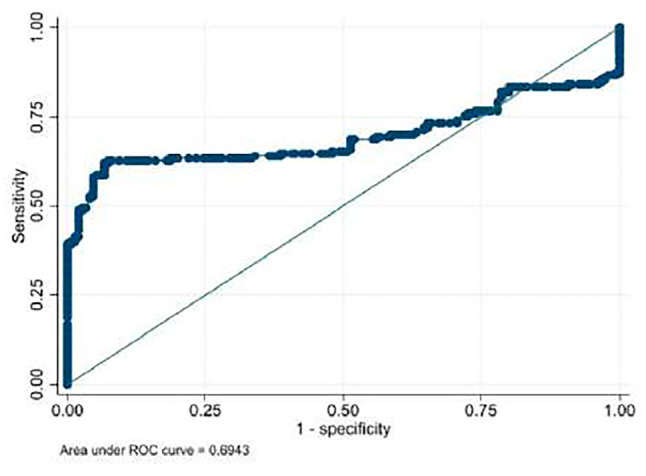
ROC curve for P53 for predicting the cases. As illustrated in Figure 5, the ROC curve analysis yielded an AUC of 0.6943, suggesting modest diagnostic accuracy of the evaluated biomarker, with limited sensitivity and specificity for distinguishing between case and control groups.

Figure 6 presents the results of the ROC analysis for predicting P53 gene mutation cases. The Area Under the Curve (AUC) is 0.6943, which indicates that the P53 gene mutation has a moderate ability to distinguish between individuals with and without the condition. While the AUC is above 0.5, suggesting some predictive value, it is not an exceptionally strong predictor. The Z statistic of 5.23 and the significance level of *p*<0.0001 indicate that the AUC is statistically significant, meaning the P53 gene mutation is a relevant marker in distinguishing between the two groups.

Table 3 highlights that inflammatory and prostate-related markers significantly influence P53 gene expression. IL-6 (*p*=0.007) and PSA (*p*=0.043) showed significant positive associations, indicating that systemic inflammation and prostate alterations may upregulate P53 in infertile men. SOD and testosterone showed non-significant negative trends, suggesting potential roles of oxidative stress and hormonal imbalance in modulating P53 expression.

**Table 3:** Results of multiple linear regression analysis of biochemical and hormonal markers on P53 gene mutation.

Predictor Variable	Coefficient	Std. Error	t-value	p-value	95% Confidence Interval
Superoxide Dismutase (U/mL)	-0.0233	0.0226	-1.03	0.305	-0.0680 to 0.0215
Testosterone (ng/dL)	-0.0008	0.0005	-1.62	0.108	-0.00169 to 0.00017
Follicle Stim. Hormone (FSH, mIU/mL)	0.0087	0.0064	1.35	0.180	-0.0040 to 0.0214
Estradiol (pg/mL)	-0.0044	0.0046	-0.95	0.342	-0.0135 to 0.0047
Prostate Specific Antigen (ng/mL)	0.1172	0.0573	2.04	0.043	0.0038 to 0.2306
Interleukin-6 (pg/mL)	0.0346	0.0126	2.75	0.007	0.0097 to 0.0594
Constant	2.3510	0.6927	3.39	0.001	0.9806 to 3.7215

Multivariate linear regression analysis was performed to assess the relationship between biochemical and hormonal predictors and the dependent variable. Coefficients represent the change in the outcome variable for each unit increase in the predictor. Statistically significant associations were observed for prostate-specific antigen (PSA; *p*=0.043) and interleukin-6 (IL-6; *p*=0.007), with 95% confidence intervals not crossing zero. Other variables, including superoxide dismutase (SOD), testosterone, follicle-stimulating hormone (FSH), and estradiol, did not show significant predictive value (*p*>0.05), as shown in Table 3.

In contrast, NRF2 gene expression, a key regulator of antioxidant defense, showed a significant positive association with SOD (*p*<0.01), reflecting its activation in response to oxidative stress. However, IL-6 exhibited a significant negative association with NRF2 (*p*<0.05), suggesting that inflammation may suppress antioxidant gene activity. Testosterone and FSH showed weak, non-significant associations with NRF2.

**Figure 7:**
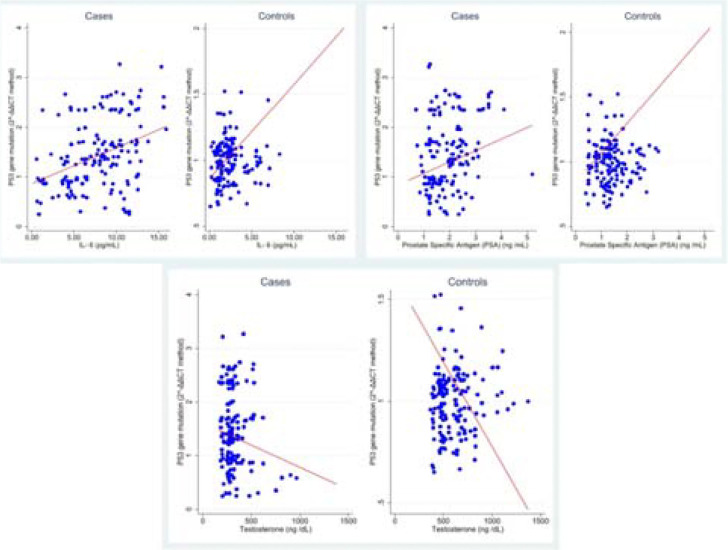
Scatterplot showing the relationship between biomarkers and P53 gene mutation expression in cases and controls.

Figure 7 illustrates the scatterplots depicting the relationship between selected biomarkers and P53 gene mutation expression in both infertile males (cases) and fertile controls. Each plot represents individual data points, allowing visual assessment of trends and differences across groups. Notably, a positive correlation is observed between IL-6 and P53 expression, indicating that elevated inflammation may upregulate this tumor suppressor gene in infertile men. Similarly, PSA levels show a significant positive trend with P53, suggesting prostate involvement. In contrast, testosterone displays inverse trends, implying that hormonal levels may contribute to increased P53 activation, possibly as a cellular stress response.

**Figure 8:**
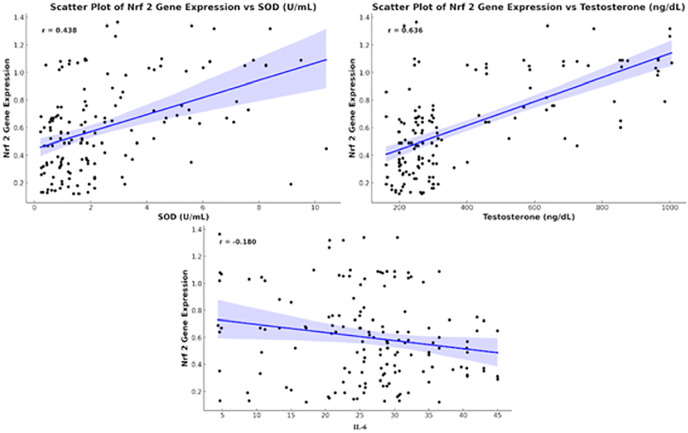
Scatterplot showing the relationship between biomarkers and NRF2 gene expression in cases and controls.

Figure 8 presents scatterplots showing the association between biomarkers and NRF2 gene expression in cases and controls. A clear positive association is evident between SOD and NRF2, supporting the role of NRF2 in regulating antioxidant defense. Similarly, a positive association is evident between Testosterone and NRF2. Conversely, a negative relationship with IL-6 suggests that inflammation may downregulate NRF2 expression. These plots help visualize how NRF2 responds differently compared to P53, highlighting the contrasting regulatory roles of oxidative stress and inflammation on these two genes in the context of male infertility.

## DISCUSSION

This case-control study investigated the biochemical and molecular alterations in infertile males, with a focus on oxidative stress, inflammation, hormonal imbalance, and gene expression patterns. The findings revealed marked differences between infertile men and fertile controls, indicating that male infertility is a multifactorial condition driven by a complex interplay of systemic and testicular stressors.

A key observation in the case group was the significant reduction in superoxide dismutase (SOD) activity and NRF2 gene expression (Table 1), indicating a compromised antioxidant defense system. SOD is a critical enzymatic antioxidant that catalyzes the dismutation of superoxide radicals into hydrogen peroxide, thereby mitigating oxidative damage to lipids, proteins, and DNA. Reduced SOD activity can impair sperm integrity and function, which is a characteristic feature of idiopathic male infertility (Agarwal *et al*., 2021). NRF2 (Nuclear Factor Erythroid 2-Related Factor 2) serves as a central transcriptional regulator of the cellular antioxidant response by modulating the expression of various cytoprotective and detoxifying enzymes (Bisht *et al*., 2017). The present study revealed a significant positive correlation between SOD activity and NRF2 expression (*p*<0.01) (Table 1), supporting the notion that NRF2 regulates antioxidant defense pathways in testicular tissues.

These findings are consistent with those of Dorostghoal *et al*. (2017), who reported markedly decreased SOD levels in infertile males, implicating oxidative stress as a key factor in male infertility. Furthermore, Pedruzzi *et al*. (2015) and Gan & Johnson (2014) have demonstrated NRF2 downregulation in various oxidative stress-related disorders, underscoring its role in maintaining cellular redox homeostasis. In the context of male infertility, impaired NRF2 signaling may lead to reduced activation of antioxidant response elements (AREs), thereby diminishing the transcription of genes involved in neutralizing oxidative insults. Together, these results highlight the pivotal role of NRF2-regulated antioxidant mechanisms, including SOD activity, in maintaining male reproductive function, and suggest that their dysregulation may significantly contribute to infertility through increased oxidative damage.

Moreover, the negative association between IL-6 and NRF2 expression (*p*<0.05) (Figure 8) indicates that chronic inflammation may inhibit NRF2 activation, thereby exacerbating oxidative stress. This aligns with findings from Dutta *et al*. (2021), who demonstrated that sustained inflammatory signaling can impair NRF2 translocation and reduce the expression of downstream antioxidant genes. Hence, infertile men in this study appear to suffer from a compounded deficit: reduced antioxidant activity and unchecked inflammation.

Inflammatory markers, especially interleukin-6 (IL-6), were significantly elevated in infertile males compared to controls (*p*<0.001) (Table 2). IL-6 is known to impair spermatogenesis by disrupting Sertoli cell function, damaging the blood-testis barrier, and triggering apoptosis of germ cells (Białas *et al*., 2009). The positive correlation between IL-6 and p53 gene expression (Figure 7) observed here suggests that inflammatory stress can activate apoptotic signaling via p53, a key tumor suppressor gene and DNA damage sensor (Hofseth *et al*., 2003). Overexpression of p53 has been associated with increased germ cell apoptosis and poor sperm quality, further supporting its role as a mediator of inflammation-induced testicular dysfunction.

Additionally, PSA (Prostate-Specific Antigen) levels were significantly higher in infertile males and showed a positive correlation with p53 expression (Figure 7). Elevated PSA may reflect subclinical prostatic inflammation or epithelial dysfunction, which can indirectly influence testicular health and male fertility (Elzanaty *et al*., 2016). The concurrent upregulation of p53 in this context further supports the idea that prostate stress and systemic inflammation may synergize to promote apoptosis in the reproductive tract.

Hormonal imbalance was also evident in infertile participants in the present study. Increased FSH and estradiol levels and reduced testosterone are characteristic of primary testicular failure and endocrine disruption. Elevated FSH is often a compensatory response to impaired Sertoli cell activity or reduced spermatogenic output (Santi *et al*., 2020). Low testosterone impairs spermatogenesis and is associated with increased oxidative and apoptotic stress. At the same time, excess estradiol, often due to enhanced aromatase activity in adipose or testicular tissue, can further suppress gonadotropin secretion and disrupt spermatogenic signaling (Jayasena *et al*., 2019). The positive association between testosterone and NRF2, and negative trend with p53, observed in this study, reinforces the role of testosterone as a protective factor against oxidative and apoptotic damage (Figures 7 and 8).

Diagnostic performance analysis using ROC curves revealed that FSH and NRF2 gene expression were the strongest predictors of infertility status, both showing high AUC values (Figure 2). These markers effectively discriminated between infertile and fertile males, with FSH reflecting testicular functional status and NRF2 serving as a novel indicator of antioxidant defense integrity. Similarly, IL-6 and estradiol showed excellent AUC values (0.8996 and 0.8875, respectively), confirming their roles as diagnostic markers of inflammation and hormonal imbalance (Figures 3 and 5). In contrast, testosterone, SOD, and p53 had moderate predictive value (AUC 0.69-0.73), suggesting that they may be more useful as supportive rather than standalone markers (Figures 2 and 6).

Scatterplot analyses provided further insights into these associations. Positive trends were observed between IL-6 and p53, as well as between PSA and p53, suggesting a pathway through which inflammation and prostate stress upregulate apoptotic responses. Conversely, SOD and testosterone positively correlated with NRF2 expression, while IL-6 showed an inverse association, highlighting the antagonistic roles of oxidative defense and inflammatory activation in shaping gene expression in infertile men.

### Limitations

Given the case-control design, causality cannot be established. The absence of semen quality parameters limits functional correlation. Future studies with longitudinal designs, inclusion of sperm DNA integrity measures, and intervention-based approaches are warranted to validate the clinical applicability of these biomarkers.

## CONCLUSION

The integrated dysregulation of NRF2 and p53 gene expression, coupled with hormonal and biochemical imbalances, plays a significant role in the pathogenesis of male infertility. These dual molecular markers may serve as valuable tools for early diagnosis and targeted therapy. Future studies should investigate the longitudinal effects and intervention strategies aimed at restoring redox balance and hormonal regulation.
